# Musculoskeletal disorders and the physical activity of territorial army soldiers during the COVID-19 pandemic

**DOI:** 10.1186/s12891-021-04654-2

**Published:** 2021-09-16

**Authors:** Małgorzata Grabara, Ewa Sadowska-Krępa

**Affiliations:** Institute of Sport Sciences, Jerzy Kukuczka Academy of Physical Education, Katowice, Poland

**Keywords:** Musculoskeletal symptoms, Occupational physical activity, Leisure time physical activity, seven-day physical activity recall, COVID-19

## Abstract

**Background:**

The aim of this study was to assess the occurrence of self-reported musculoskeletal disorders (MSD) among Polish territorial army soldiers during the COVID-19 pandemic and to investigate whether there was a relationship between occupational physical activity (OPA), leisure time physical activity (LTPA), and MSD.

**Methods:**

The study used a cross-sectional design with a sample of 373 territorial army soldiers ages 18–55 who had not previously suffered from COVID-19 and were not convalescents. The symptoms prevalence data was collected using the standardized Nordic Musculoskeletal Questionnaire. OPA and LTPA data was collected using the Seven-Day Physical Activity Recall (SDPAR).

**Results:**

The OPA, LTPA, and total physical activity (PA) among the studied soldiers was very diverse and the mean level of PA was relatively high. A total of 56 and 40% of territorial army soldiers had experienced pain or other discomfort in one or more of nine body regions during the past 12 months and during the past 7 days, respectively. The most common MSD among Polish territorial army soldiers were low back pain, followed by pain in the neck and knees.

**Conclusions:**

The study revealed that the OPA of the studied soldiers, especially vigorous-intensity and high vigorous-intensity OPA, was associated with a higher prevalence of MSD in several regions of the body, i.e. the lower back, elbows, wrists or hands, hips or thighs, and ankles or feet. Along with the increase in energy expenditure on total PA, a greater percentage of respondents experienced low back pain. Vigorous and high vigorous-intensity PA may contribute to the occurrence of MSD.

## Background

Musculoskeletal disorders (MSD) defined as self-reported musculoskeletal symptoms are prevalent in working populations worldwide and cause major health issues [1, 2]. Disorders of the muscles, tendons, joints, and spinal discs may be caused by work and lead to a decrease in work effectiveness and quality of life [3, 4]. Prolonged sitting and insufficient physical activity (PA) may lead to a high risk of MSD. Previous studies also involving the military population have suggested that age, tall body height, low aerobic fitness and endurance, extremes in flexibility, prior injury, participation in recreational sports activity, a history of prior limited PA, and even older running shoes are risk factors for developing MSD [5–8].

The beneficial effects of PA are well documented, however this documentation is restricted to leisure time physical activity (LTPA). Emerging studies have shown that this effect differs depending on the domain of the PA [9], i.e. occupational physical activity (OPA), household PA, active transport, and LTPA. Several studies have demonstrated that OPA is not beneficial for health due to its too low intensity, too long duration, static and constrained postures, and insufficient recovery time [10, 11]. Moreover, the dose of PA is also important. Greater intensities and volumes of exercise may lead to greater the risk of injury and harm, specially musculoskeletal [12]. Based on WHO recommendations, a longer duration of moderate-intensity PA leads to achieving the same health benefits as shorter durations of vigorous-intensity PA [13].

PA is described as a benefit for workers in the context of decreasing the risk of musculoskeletal disorders. Previous studies have indicated that PA can prevent and reduce the occurrence of MSD regardless of age [2, 14–16]. However, a majority of them were limited to the assessment of LTPA. There is limited knowledge about PA as a factor in preventing MDS, and studies on the association between PA and MSD reveal inconsistent results [17, 18]. Physical training may also be a source of musculoskeletal injuries.

The role of the territorial defense forces in Poland is crisis prevention. During the COVID-19 pandemic their role has been special, although the training of soldiers is different than during a non-pandemic period. Considering the commonly known negative effects of the pandemic on citizen [19], and health [20, 21], the main role of the territorial defense forces was to provide comprehensive service for the civilian population. From January 2020 to August 2021, Poland submitted to the WHO [22], 2,883,120 confirmed cases of COVID-19 with 75,261 deaths.

Service in the territorial armies is voluntary and the number of vocations depends on the capabilities and availability of the volunteers, who are professionally active and represent different levels of PA. However, all soldiers are obliged to maintain their physical fitness individually, with the army providing them the opportunity to use the gym and improve their swimming skills at a swimming facility up to 2 h a week. They are required to pass an annual fitness test. The necessity of undertaking PA among soldiers could have an influence on MSD.

The aim of this study was to assess the occurrence of self-reported musculoskeletal disorders among male and female territorial army soldiers during the COVID-19 pandemic, and to investigate whether there was a relationship between occupational physical activity, leisure time physical activity, and MSD.

## Methods

### Study design

A cross-sectional survey research of male and female Polish territorial army soldiers was performed. This study was approved by the Bioethics Committee of the Jerzy Kukuczka Academy of Physical Education in Katowice (certificate of approval No. KB/02/12) and conformed to the standards established by the Declaration of Helsinki. All participants were told about the type and purpose of the study and gave their informed consent prior to filling out the questionnaire.

### Participants

The study recruited a sample of 373 territorial army soldiers ages 18–55 years who had not previously suffered from COVID-19 and were not convalescents. However, it should be noted that participants were not tested, therefore if they were asymptomatic, they may have been infected without knowing.

The studied sample included all soldiers from one of the fifteen Territorial Defense Brigades in Poland who had completed the entire training cycle and were authorized to perform tasks related to their official duties. The inclusion criteria were: a) possession of a medical certificate of fitness to serve in the territorial army, b) successful completion of fitness examinations, c) at least 6 months of service in the territorial army, d) the consent to participate in the study. Participants who did not meet the inclusion criteria as well as those with missing data were excluded. The basic characteristics of the studied soldiers are presented in Table 1.

**Table 1:** Basic characteristic of the participants, declared time spent sitting per day, and energy expenditure (EE) on total physical activity (PA) during the week [kcal]

Variables	N	%
Gender		
Men	305	82%
Women	68	18%
Age [years]		
< 25	85	23%
25–40	212	57%
> 40	76	20%
BMI		
Underweight	8	2%
Healthy weight (norm)	169	45%
Overweight	159	43%
Obese	37	10%
Time spent sitting per day		
< 2 h	47	13%
2–4 h	73	19%
4–6 h	129	35%
6–8 h	68	18%
above 8 h	56	15%
EE on total PA		
< 2000 kcal	87	23%
2000–3999 kcal	66	18%
4000–6000 kcal	70	19%
> 6000 kcal	150	40%

### Methods and procedures

The study was conducted from October–November 2020 using a direct pen-and-paper interview method in the workplace of the studied participants.

The symptoms prevalence data was collected using the standardized Nordic Musculoskeletal Questionnaire (NMQ). NMQ contains a view of the human body imaged from the back and separated into the nine anatomical localizations which may be affected by musculoskeletal disorders. The NMQ quantifies musculoskeletal pain in the following body regions: neck, shoulders, upper back, elbows, low back, wrists/hands, hips/thighs, knees, and ankles/feet. The participants provided information related to their pain symptoms (discomfort, numbness, ache) during the last 12 months and during the last 7 days. In case of pain complaints during the last 7 days, an estimation of the pain intensity was given using a range of 1 to 10 (from a minimal to intense or unbearable pain) [23–25].

Body mass index was estimated on the basis of the participants’ body height and weight.

Occupational and leisure time physical activity data was collected using the Seven-Day Physical Activity Recall (SDPAR) with the authors’ own modification. The questionnaire serves for collecting data concerning the frequency, intensity, and duration of both occupational and leisure time PA as well as the time spent sleeping seven days prior to examination. Domestic PA and active transport (commuting PA) were included in leisure time PA, whereas active transport to work was included in occupational PA due to the sparse reporting of these activities by the studied soldiers (by four participants or less).

Participants declared their occupational physical activity (OPA) and leisure time physical activity (LTPA) separately (in minutes) for each day of the week, describing its intensity as moderate, vigorous, or highly vigorous, as well as the number of hours spent sleeping. They also declared the mean time spent sitting per day. The declared PA was estimated according to the SDPAR procedure: moderate-intensity PA – 4 METs, vigorous-intensity PA – 6 METs, high-vigorous-intensity PA – 10 METs [26, 27]. Taking all the data into consideration, weekly and daily energy expenditures on PA were calculated [kcal, METmin].

### Statistical analysis

Results are expressed as the means, standard deviations, medians, minimal and maximal values, and confidence interval (95%) of standard deviations or are described using frequencies (percentage). The normality of distribution was verified with the Shapiro-Wilk test. To compare the number of soldiers within each category of energy expenditure (EE) on total PA, classified as < 2000 kcal, 2000–3999 kcal, 4000–6000 kcal, and > 6000 kcal, and time spent sitting classified as < 2 h, 2–4 h, 4–6 h, 6–8 h, above 8 h, and BMI status classified as underweight (< 18.5), healthy weight (18.5–24.9), overweight (25.0–29.9), and obese (≥ 30.0) with the number of workers with MSD (separately for each body region), Pearson’s chi-squared test was used. To determine the relationship between PA and the prevalence of MSD, Spearman’s rank correlation coefficient and multiple regression were applied. To compare PA (total as well as domain and intensity specific) between the soldiers who reported MSD classified as a lack of MSD, 1 or 2 MSD, or 3 or more MSD, the Kruskal-Wallis one-way ANOVA by ranks or one-way ANOVA was used. The level of significance was set at *p* ≤ .05. Statistical analysis was undertaken using Statistica ver. 13, TIBCO Software Inc.

## Results

The basic characteristic of participants regarding their sex, age, BMI, time sent sitting per day, and EE on total PA is shown in Table 1.

The occurrence of MSD during the last 12 months and the last 7 days is presented in Table 2. The 12-month prevalence of low back pain was 36%, knee pain 22.5%, and neck pain 21%. The last 7-day prevalence of MSD in certain body regions was lower than the last 12 months. The highest average pain intensity was reported for the low back and knees (Table 2).

**Table 2:** The occurrence of musculoskeletal disorders (MSD) during the last 12 months and during the last 7 days, pain intensity of MSD during the last 7 days among soldiers (*n* = 373)

Area of body affected	Occurrence of MSD during last 12 months [n, %]	Occurrence of MSD during last 7 days [n, %]	Pain intensity (1–10) of MSD during last 7 days (min-max)	Pain intensity (1–10) of MSD during last 7 days (mean ± sd)
**Neck**	78 (21%)	43 (12%)	1–9	4.07 ± 2.21
**Shoulders**	49 (13%)	31 (8%)	1–8	4.23 ± 1.90
**Upper back**	52 (14%)	36 (10%)	2–10	4.56 ± 2.28
**Elbows**	18 (5%)	10 (3%)	2–6	4.30 ± 1.10
**Wrists/hands**	39 (20%)	20 (5%)	1–8	3.85 ± 1.90
**Lower back**	134 (36%)	72 (19%)	1–10	4.79 ± 1.94
**Hips/thighs**	33 (9%)	24 (6%)	1–8	4.25 ± 2.03
**Knees**	84 (23%)	44 (12%)	1–10	4.73 ± 2.49
**Ankles/feet**	46 (12%)	33 (9%)	1–10	2.47 ± 2.21

The occupational, leisure time, and total PA among studied soldiers was very diverse and the mean level of PA was relatively high (Table 3). As many as 40% of participants spent more than 6000 kcal per week on their total PA, and only 15% of soldiers declared more than 8 h spent sitting per day (Table 1).

**Table 3:** Descriptive statistics of physical activity (PA) among soldiers (n = 373)

Weekly PA	Mean ± SD	Median	Min-Max	SD (CI_L_ - CI_U_)
OPA Moderate [METmin]	2404.9 ± 2595.87	1440	0–10,080	2422.0 ± 2796.8
OPA Vigorous [METmin]	1189.5 ± 1491.22	360	0–6480	1391.3 ± 1606.7
OPA High vigorous [METmin]	545.8 ± 1064.69	0	0–6000	993.4 ± 1147.1
OPA Total [METmin]	4140.3 ± 4155.71	2600	0–14,880	3877.4 ± 4477.5
OPA Total [kcal]	5588.1 ± 5749.32	3499	0–22,050	5364.2 ± 6194.4
LTPA Moderate [METmin]	1962.7 ± 2047.36	1400	0–10,080	1910.2 ± 2205.9
LTPA Vigorous [METmin]	1084.7 ± 1203.76	720	0–5040	1123.1 ± 1297.0
LTPA High vigorous [METmin]	702.4 ± 1099.63	0	0–4300	1026.0 ± 1184.8
LTPA Total [METmin]	3749.8 ± 3458.27	3000	0–19,320	3226.6 ± 3726.0
LTPA Total [kcal]	4953.3 ± 4691.22	3956	0–25,760	4377.0 ± 5054.4
PA (OPA + LTPA) Total [METmin]	7890.2 ± 6463.12	6440	0–29,060	6030.2 ± 6963.5
PA (OPA + LTPA) Total [kcal]	6063.3 ± 5101.43	4964	0–24,909	4759.7 ± 5496.4

Legend: OPA – occupational physical activity; LTPA – leisure time physical activity; CI – confidence interval (95%); Total = moderate + vigorous + highly vigorous PA

Based on energy expenditures on total PA during the week (as shown in Table 1), the analysis showed statistically significant differences in the prevalence of low back pain (Pearson χ^2^ = 8.13, *p* = .043). Other differences were not observed.

Based on declared time spent sitting per day and BMI status (as shown in Table 1), the analysis did not reveal any significant differences in the prevalence of MSD.

**Fig. 1:**
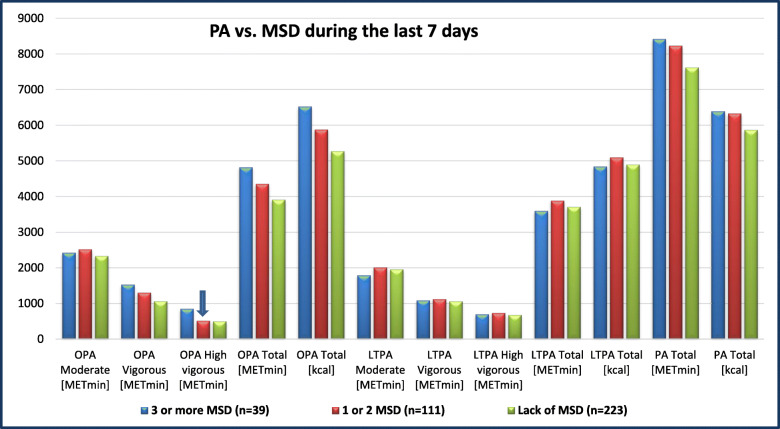
The level of OPA, LTPA, and total PA vs. the occurrence of MSD during the last 7 days

**Fig. 2:**
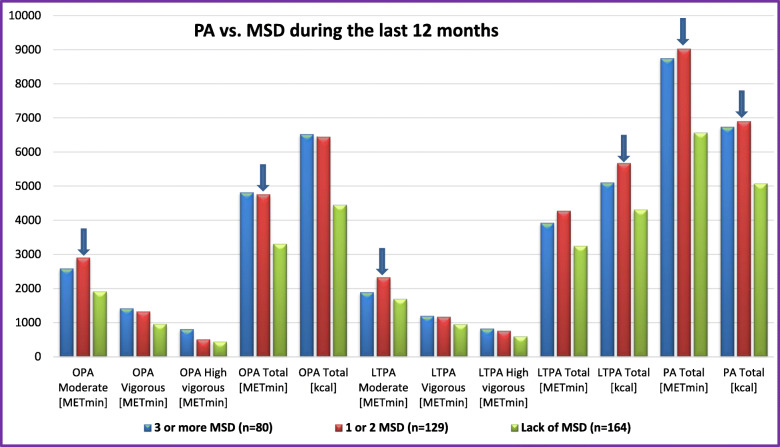
The level of OPA, LTPA, and total PA vs. the occurrence of MSD during the last 12 months

The association between PA and the occurrence of MSD during the last 7 days and during the last 12 months is presented in Figs. 1 and 2, respectively. The analysis showed a difference in high vigorous-intensity OPA (*p* = .019) between soldiers who did not report any MSD during the last 7 days, those who reported 1–2 MSD, and those who reported 3 or more MSD during the last 7 days. Differences in moderate-intensity OPA (*p* = .006), total OPA (*p* = .008), moderate-intensity LTPA (*p* = .028), total LTPA (*p* = .021), total PA [METmin] (*p* = .023), and total PA [kcal] (*p* = .003) were found between soldiers who reported no MSD, those who reported 1–2 MSD, and those who reported 3 and more MSD during the last 12 months.

The following relationships between the prevalence of MSD during the last 12 months and PA were observed: the occurrence of elbow pain was correlated with vigorous-intensity OPA (r = 0.10, *p* = .044), the occurrence of wrist or hand pain was correlated with high vigorous-intensity LTPA (r = 0.11, *p* = .033), the occurrence of low back pain was correlated with moderate-intensity OPA (r = 0.17, *p* < .0001), high vigorous-intensity OPA (r = 0.11, *p* = .029), total OPA [METmin] (r = 0.16, *p* = .001), total OPA [kcal] (r = 0.17, *p* = .0001), total PA [METmin] (r = 0.15, *p* = .004), and total PA [kcal] (r = 0.14, *p* = .006).

The following relationships were observed between the prevalence of MSD during the last 7 days and PA: the occurrence of elbow pain was correlated with vigorous-intensity OPA (r = 0.13, *p* = .015), the occurrence of wrist or hand pain was correlated with high vigorous-intensity OPA (r = 0.12, *p* = .017), the occurrence of low back pain was correlated with high vigorous-intensity OPA (r = 0.14, p = .006), the occurrence of hip or thigh pain was correlated with high vigorous-intensity OPA (r = 0.13, p = .015), and the occurrence of ankle or feet pain was correlated with vigorous-intensity OPA (r = 0.13, *p* = .013) and high vigorous-intensity OPA (r = 0.17, *p* = .001). However, these correlations were weak.

The regression model explained only 3% of the variance of low back pain symptoms. It was a coincidence that with an increase in OPA moderate, the incidence of low back pain symptoms during the last 12 months actually slightly increased. The other parameters included in this model (OPA vigorous, OPA high vigorous, OPA total, and gender) were statistically insignificant. Different models were not found.

## Discussion

The aim of this study was to assess the occurrence of self-reported MSD among male and female territorial army soldiers during the COVID-19 pandemic and to investigate whether there was a relationship between OPA, LTPA, and MSD.

An investigation on the 12-month and 7-day prevalence of MSD revealed that the most common was low back pain, followed by neck and knee pain. A total of 56 and 40% of territorial army soldiers had experienced pain or other discomfort in one or more of nine body regions during the past 12 months and during the past 7 days, respectively. It should be emphasized, however, that these pain symptoms were not related to COVID-19, while myalgia, defined as muscle aches and pain, is a common clinical feature of COVID-19 [28]. Morken et al. in their study of the Royal Norwegian Navy observed that 85% of these soldiers had experienced MSD, and the most common MSD were also in the lower back, shoulders, and neck. The authors noticed that civilians had a higher prevalence of MSD than military personnel [1]. In Serra et al., police officers often reported pain in the lower back (47%) and dorsal region (33%) [29].

We observed relatively high and highly diverse PA among territorial army soldiers. Most of them declared EE on total PA above 6000 kcal peer week, and the mean and median of the total PA in the studied soldiers was above 6000 METmin per week.

In this cross-sectional study, we investigated the relationship between PA and MSD among soldiers. The study revealed that occupational PA, especially vigorous-intensity and high vigorous-intensity OPA, is positively correlated with a higher prevalence of MSD in several regions of the body, i.e. the lower back, elbows, wrists or hands, hips or thighs, and ankles or feet. We observed that along with an increase in energy expenditure on total PA, a greater percentage of respondents experienced low back pain. We also observed that undertaking high vigorous-intensity LTPA is positively correlated with a higher prevalence of wrist or hand pain. Our study indicates that participants who were less physically active experienced MSD less often during the last 12 months. This probably results from the declared high levels of PA among most of the studied soldiers. Our findings are partially in line with previous studies.

As showed in Weyh et al., welders who had a higher physical work load demonstrated a higher prevalence of MSD. The 12-month prevalence of low back pain in welders was 71%, neck pain 61%, and shoulder pain 55%. The authors stated that insufficient LTPA (< 600 MET/week) was associated with low back pain [30].

López-Bueno et al., based on their study including 10,427 active Danish employees, observed an association between LTPA and long-term sickness absence (LTSA). The authors noticed that high LTPA reduced the risk of LTSA by a borderline significant 23%, whereas moderate LTPA also reduced LTSA but a lower percentage than high LTPA [31].

Søgaard et al. revealed that employees with high work activity had a higher frequency of recurring lower back and hand/wrist symptoms, whereas employees with mainly sitting work experienced a higher frequency of recurrent neck symptoms [32]. Our findings were similar; however, we did not find an association between time spent sitting per day and the prevalence of MSD, including neck pain.

The comparison of OPA, LTPA, and MSD between physical education teachers (PET) and other teachers indicated that PET who were more physically active had a significantly lower risk of all MSD during the past year then teachers who were more sedentary [33]. These findings point out that long-term PA is associated with a lower risk of MSD, which is not in line with our study. This is likely due to different levels of PA between teachers and soldiers.

Due to the demands of the annual fitness test, soldiers are obliqued to maintain high fitness levels, which are related to performing physical training. For the same reason, this occupational group might differ from other working groups and should not be compared directly.

The lack of the positive effects of PA, the relationship between vigorous and high-vigorous PA, and the occurrence of MSD in the hips or thighs or ankles or feet observed in our study is supported by studies that indicate physical training as the cause of injuries in the lower limbs [34, 35].

The opposite findings were obtained by Serra et al. who indicated that undergoing physical activities during the last 12 months reduced the odds of getting MSD by 30%. Police officers who had performed PA during the last 12 months reported less occupational stress than those who did not. However, the authors mainly focused on the assessment of stress perception, whereas information about PA was limited to one question regarding performing some type of physical exercise more than three times a week [29].

Our results, based on the cross-sectional study design, do not allow us to point out a clear causal relationship between PA and MSD. However, we may assume that performing high-intensity PA, the majority as OPA, causes the more frequent occurrence of MSD.

### Strengths and limitations of the study

The study was conducted during the COVID-19 pandemic among a specific group of workers with relatively high PA. These results should neither be generalized to other working populations nor to non-pandemic periods.

There are several limitations to this study which are worth mentioning. In this study, PA was measured by a questionnaire, a subjective method of assessing PA, and the validity and reliability of this method may be questionable [36]. As has been shown in previous studies, self-reported PA is often overestimated [37, 38]. However, a questionnaire may be the one practical method of evaluating PA among a large population. Another limitation is not distinguishing between domestic PA and active travel as domains of PA due to the sparse reporting of these activities, whereas participants reported occupational and leisure time PA separately. Further, another limitation is the lack of information about education, social status, and physical fitness. However, as was shown in the inclusion criteria, a fairly homogeneous group was examined. We assumed that the physical fitness of the studied soldiers would be similar due to the requirement to pass obligatory annual fitness tests. On the other hand, this study also included the following potential confounders: age, gender, education.

## Conclusions

The most common MSD among Polish territorial army soldiers were low back pain, followed by neck and knee pain. Participants had relatively high and highly diverse levels of PA.

The study revealed that the OPA of the studied soldiers, especially vigorous-intensity and high vigorous-intensity OPA, was positively correlated with a prevalence of MSD in several regions of the body, i.e. the lower back, elbows, wrists or hands, hips or thighs, ankles or feet. Along with the increase in energy expenditure on total PA, a greater percentage of respondents experienced low back pain.

Vigorous and high vigorous-intensity PA may contribute to the occurrence of MSD.

## Data Availability

Data and publication materials are available upon request from M.G. (m.grabara@awf.katowice.pl).

## Abbreviations

PA: physical activity
MSD: musculoskeletal disorders
OPA: occupational physical activity
LTPA: leisure time physical activity
SDPAR: Seven-Day Physical Activity Recall
NMQ: Nordic Musculoskeletal Questionnaire
EE: energy expenditure
BMI: body mass index
